# Serum HMGB1 level is correlated with serum I-FABP level in neonatal patients with necrotizing enterocolitis

**DOI:** 10.1186/s12887-021-02818-6

**Published:** 2021-08-21

**Authors:** Ruyahan Huo, Heng Liu, Jing Chen, Hong Sheng, Li Miao

**Affiliations:** 1 Department of Neonatology, The Affiliated Lianyungang Hospital of Xuzhou Medical University, The First People’s Hospital of Lianyungang, 222000 Lianyungang, China; 2 Department of Pediatrics, The Affiliated Lianyungang Hospital of Xuzhou Medical University, The First People’s Hospital of Lianyungang, No. 182 Tongguan North Road, 222000 Lianyungang, China

**Keywords:** HMGB1, I-FABP, Neonates, Necrotizing enterocolitis

## Abstract

**Background:**

This study aims to investigate clinical significance of HMGB1 in neonatal patients with necrotizing enterocolitis (NEC).

**Methods:**

This observational study enrolled a total of 106 stage II-III NEC neonatal patients, who were admitted in our hospital from March 2014 to March 2019. In addition, 99 suspected NEC patients and 200 healthy controls were included. The serum levels of HMGB1, I-FABP, and inflammatory factors CRP, IL-1β, IL-6 and TNF-α were determined by enzyme-linked immunosorbent assay (ELISA). Then, the demographic data and clinical characteristics of all patients were collected. Statistical analysis was conducted to determine the correlation between HMGB1 and the clinical characteristics.

**Results:**

No significant difference was found in the basic characteristics of NEC patients and healthy controls, except for birth weight and gestational age. The expression levels of HMGB1, I-FABP, and inflammatory factors IL-1β, IL-6 and TNF-α were significantly higher in NEC patients, when compared to healthy controls. The serum levels of HMGB1, I-FABP, IL-1β and IL-6 markedly increased in stage II-III NEC patients, when compared to stage I NEC patients. The Pearson’s analysis revealed a positive correlation between HMGB1 and I-FABP, HMGB1 and IL-1β, and HMGB1 and IL-6. The ROC curve revealed that both HMGB1 and I-FABP can potentially be used as diagnostic factors for NEC. The logistic multivariate regression revealed that I-FABP, IL-1β and IL-6 are independent risk factors for mortality in neonatal NEC patients.

**Conclusions:**

Serum HMGB1 levels are upregulated in neonatal NEC patients, and these are correlated with the patient’s prognosis.

## Background

Necrotizing enterocolitis (NEC) is the most serious intestinal disease in neonates, and has an estimated incidence of 6–7 % worldwide [1]. Furthermore, NEC has a very high mortality that ranges within 10–50 %, according to different researches [2–5]. In general, the incidence of NEC is more common in premature infants, especially in neonates with very low birth weight [5, 6], and its incidence continues to increase at present [7, 8]. Since inflammation is one of the main characteristics of NEC, a number of inflammatory factors, such as C-reactive protein (CRP), IL-1β, IL-6, TNF-α and other factors, including intestinal fatty acid binding protein (I-FABP), are involved in NEC [9, 10]. Therefore, new biomarkers are needed for both the diagnosis and prognosis of NEC.

High mobility group box-1 (HMGB-1) is a newly identified inflammation-related factor. In recent years, the association between HMGB1 and inflammation has been elucidated in various studies, including intestinal diseases. In intestinal diseases, it was found that HMGB1 can promote experimental colitis in a mouse model through the regulation of IL-23 [11]. In another recent research, Chen et al. reported that HMGB1 and NLRP3 are elevated in ulcerative colitis patients, and are correlated with the patient’s clinical outcome [12]. However, to date, few studies have focused on the clinical significance of HMGB1 in neonatal patients with NEC.

The present study conducted an observational research to investigate the role of HMGB1 in neonatal NEC patients, and its relationship with the clinical outcomes of NEC patients, and the serum levels of I-FABP and inflammatory factors. It was hypothesized that HMGB1 might be abnormally expressed in NEC, and might be associated with the patient’s clinical outcome. The present study might provide more clinical data and research targets for HMGB1 in NEC patients.

## Methods

### Patients

The present observational study enrolled a total of 106 neonatal NEC patients, who were admitted in our hospital from March 2014 to March 2019. All patients were diagnosed with NEC based on their clinical symptoms (at least three or more), including unstable temperature, apnea, impotence, increased residual milk in stomach, midabdominal distension, vomiting coffee-like substances, positive stool occult blood, etc. The diagnosis of NEC was confirmed by X-ray imaging and surgical results. For severe NEC patients who received surgery, the diagnosis was intra-operatively confirmed. The severity of NEC was measured according to the modified Bell’s staging criteria [13]. Merely stage II-III patients were defined as NEC, while stage I patients were suspected with NEC. The inclusion criteria were, as follows: [1] patients within one month after birth; [2] patients diagnosed with NEC, and had no other intestinal diseases, such as congenital intestinal obstruction or Meniere’s diverticulum. The following patients were excluded: [1] patients with other severe infections; [2] patients with Hirschsprung’s disease, digestive tract malformation and other intestinal diseases; [3] patients with congenital heart, liver, or renal diseases. In addition, a total of 99 suspected NEC patients were included, and another 200 healthy neonates, who were born in our hospital, were enrolled as healthy controls. A written informed consent was obtained from the parents of all patients. The present study was approved by the Ethics Committee of The Affiliated Lianyungang Hospital of Xuzhou Medical University, The First People’s Hospital of Lianyungang.

### Measurement of serum HMGB1, I-FABP and inflammatory factors

The blood samples of all patients and healthy controls were collected within 24 h after admission. The serum levels of HMGB1, I-FABP, and inflammatory factors CRP, IL-1β, IL-6 and TNF-α were determined by enzyme-linked immunosorbent assay (ELISA) using commercial ELISA kits (HMGB1 LifeSpan Biosciences LS-F26519, I-FABP Abcam ab193700, CRP Abcam ab181416, IL-1β Abcam ab46052, IL-6 Abcam ab178013, and TNF-α Abcam, ab181421), according to manufacturer’s instructions.

### Data collection

The demographic data and clinical characteristics of all patients were collected, including age at diagnosis, gender, gestational age, birth weight, Apgar score, delivery mode, NEC stage, and surgery methods (if the patient received surgery). All patients were followed up for six months for the survival analysis.

### Statistical analysis

All data were calculated using the SPSS software, version 20.0 (IBM, Armonk, NY, USA) and GraphPad Prism 6.0 (GraphPad Software, Inc.). Continuous data were presented as mean ± standard deviation (SD). Kolmogorov-Smirnov and Shapiro-Wilk analysis were used to confirm the distribution type of the data. Comparisons between two groups was conducted by Student’s *t*-test, and comparisons among three or more groups was conducted by one-way analysis of variance (ANOVA), followed by Tukey post hoc test. Chi-squared analysis was used to analyze the rates. The correlation among HMGB1 and the inflammatory factors was analyzed by Pearson’s rank correlation analysis. The ROC curve was used for the diagnostic analysis. The cut-off value was based on both the sensitivity and specificity. *P* < 0.05 was considered statistically significant.

## Results

### Basic clinical characteristics of all patients

The present study enrolled 106 neonatal NEC patients, with a mean age at diagnosis of 47.82 ± 24.29 weeks old. As shown in Table 1, no significant difference was found in the basic characteristics of NEC patients, suspected NEC patients and healthy controls, except for birth weight and gestational age.

**Table 1:** Basic characteristics of all participants

Variables	Stage II-III NEC, *n* = 106	Suspected Stage I NEC, *n* = 99	Healthy, *n* = 200	*P*-value^*^
Age at diagnosis, weeks	47.82 ± 24.29	51.98 ± 24.39	50.09 ± 23.45	0.457
Gender, female (%)	45 (43.69)	41 (41.41)	90 (45.00 %)	0.874
Gestational age, weeks	31.04 ± 4.88	32.53 ± 4.84	36.46 ± 2.82	< 0.001
Birth weight, kg	2.74 ± 0.76	2.72 ± 0.73	3.45 ± 0.62	< 0.001
1-min Apgar score	6.97 ± 1.38	7.06 ± 1.30	6.99 ± 1.46	0.894
5-min Apgar score	8.05 ± 1.37	7.87 ± 1.41	7.97 ± 1.42	0.666
Delivery mode, *n* (%)				0.836
Vaginal delivery	64 (60.38)	62 (62.63)	117 (58.50)	
Caesarean delivery	42 (39.62)	37 (37.37)	83 (41.50)	
Surgery methods, *n* (%)				
Laparotomy	52 (49.06)	-	-	
Peritoneal Drainage	54 (50.94)	-	-	
Mortality rate, *n* (%)	19 (17.94)	-	-	

*Comparisons among three or more groups were performed by one-way analysis of variance (ANOVA), followed by Tukey’s post hoc test

### HMGB1 was upregulated in neonatal NEC patients

**Fig. 1:**
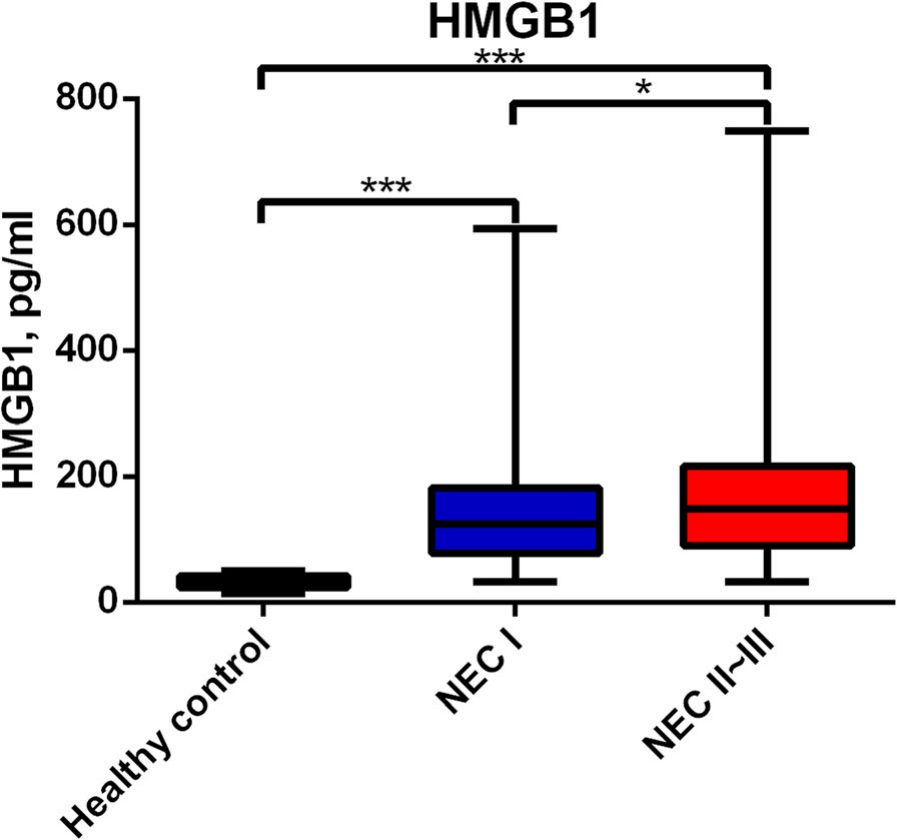
Serum levels of HMGB1 in NEC patients, suspected stage I NEC patients, and healthy controls

The serum expression levels of HMGB1 were determined for NEC patients, suspected NEC patients, and healthy controls. It was found that the serum level of HMGB1 remarkably increased in all NEC patients, when compared to healthy controls (*P* < 0.05, Fig. 1). In addition, stage II-III NEC patients had significantly higher serum levels of HMGB1, when compared to suspected stage I NEC patients (*P* < 0.05). These results suggest that HMGB1 might be associated with the NEC stage.

### HMGB1 serum levels are correlated with I-FABP and inflammatory factors

**Fig. 2:**
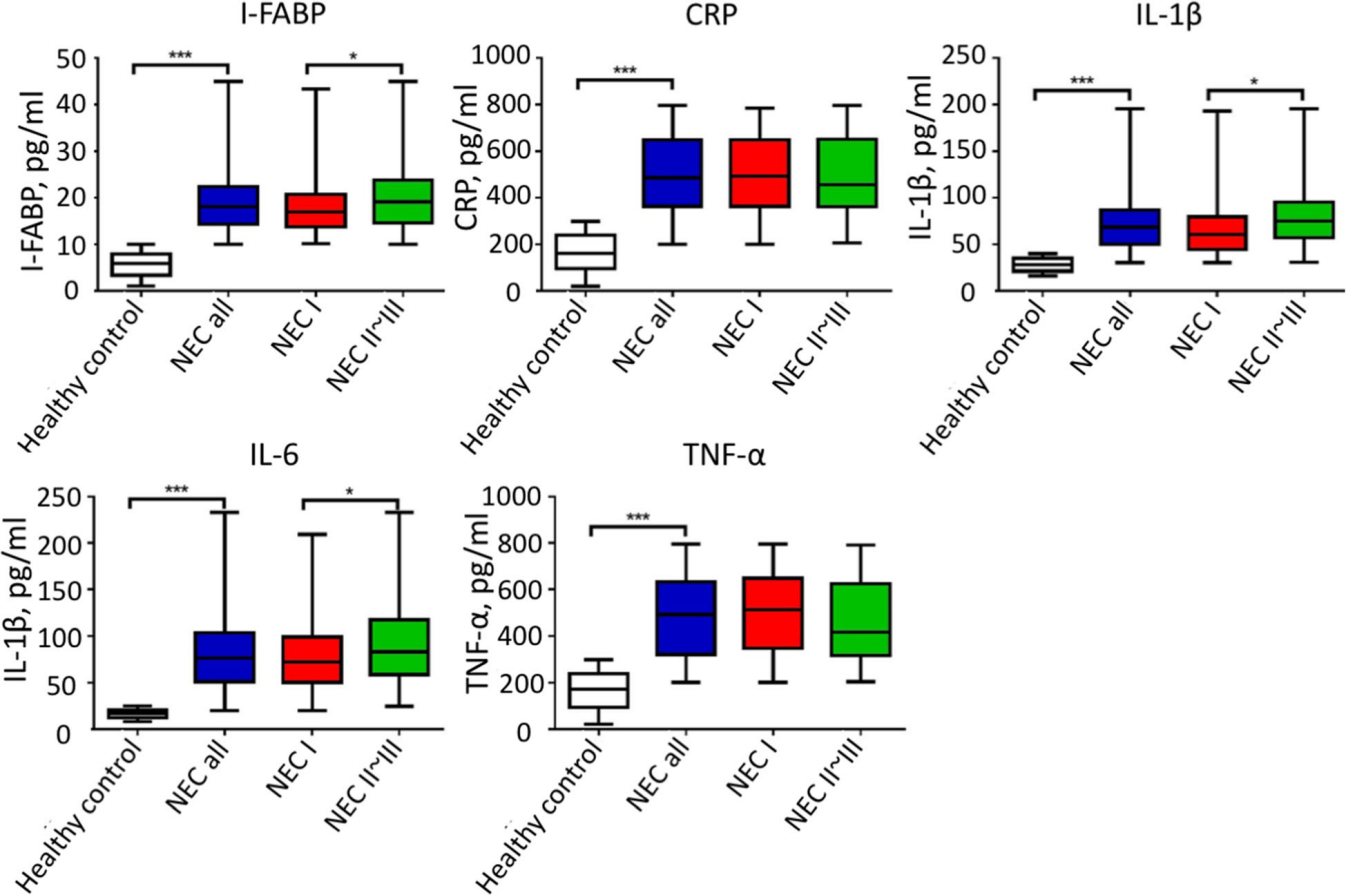
Serum levels of I-FABP and inflammatory factors in NEC patients, suspected stage I NEC patients, and healthy controls

Next, the serum levels of I-FABP and inflammatory factors CRP, IL-1β, IL-6 and TNF-α were determined in different NEC stage patients and controls. As shown in Fig. 2, the expression levels of I-FABP, and inflammatory factors IL-1β, IL-6 and TNF-α were significantly higher in NEC patients, when compared to healthy controls (*P* < 0.05). However, merely the serum levels of I-FABP, IL-1β and IL-6 markedly increased in stage II-III NEC patients, when compared to suspected stage I NEC patients. Furthermore, no significant difference was found for CRP and TNF-α in NEC patients and suspected NEC patients. Pearson’s analysis was performed to determine the correlation among HMGB1, I-FABP, and the inflammatory factors. The results revealed a positive correlation between HMGB1 and I-FABP, HMGB1 and IL-1β, and HMGB1 and IL-6 (Table 2).

**Table 2:** Pearson’s analysis for the correlation among HMGB1, I-FABP and inflammatory factors in neonatal NEC patients and suspected stage I NEC patients

		HMGB1	I-FABP	CRP	IL-1β	IL-6	TNF-α
HMGB1	Pearson correlation	1	0.511	0.001	0.282	0.292	0.026
	*P*	-	< 0.001	0.986	< 0.001	< 0.001	0.708
I-FABP	Pearson correlation	0.511	1	0.056	0.426	0.340	0.092
	*P*	< 0.001	-	0.425	< 0.001	< 0.001	0.189
CRP	Pearson correlation	0.001	0.056	1	0.109	-0.005	0.034
	*P*	0.986	0.425	-	0.119	0.946	0.624
IL-1β	Pearson correlation	0.282	0.426	0.109	1	0.383	-0.069
	*P*	< 0.001	< 0.001	0.119	-	< 0.001	0.324
IL-6	Pearson correlation	0.292	0.340	-0.005	0.383	1	-0.043
	*P*	< 0.001	< 0.001	0.946	< 0.001	-	0.541
TNF-α	Pearson correlation	0.026	0.092	0.034	-0.069	-0.043	1
	*P*	0.708	0.189	0.624	0.324	0.541	-

### Diagnostic value of HMGB1 and I-FABP for neonatal NEC

**Fig. 3:**
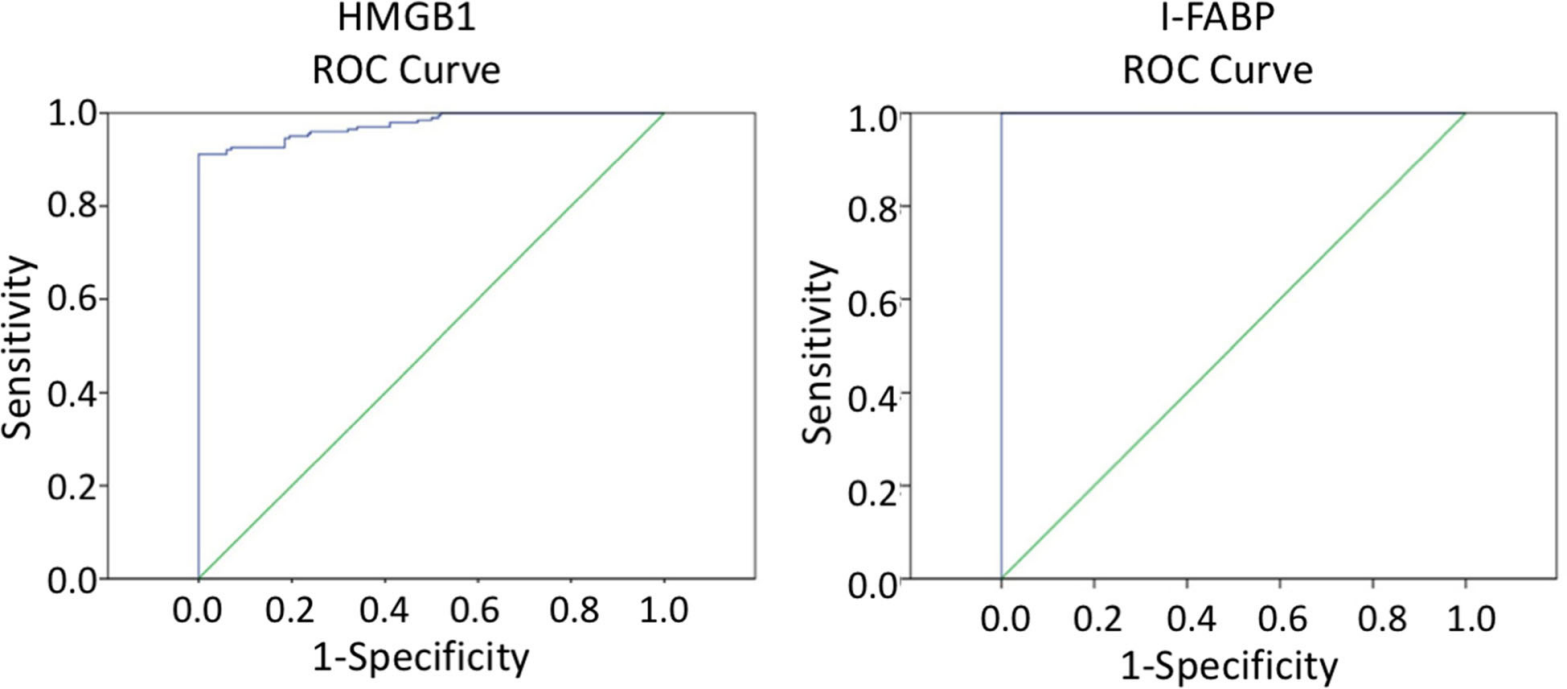
The ROC curve for HMGB1 and I-FABP in the diagnosis of neonatal NEC patients

In order to measure the diagnostic value of HMGB1 and I-FABP for NEC patients, the ROC curve was used (Fig. 3). It was found that the cut-off value for HMGB1 was 50.65 pg/ml, with an AUC of 0.852, a sensitivity of 95.3 %, and a specificity 71.2 %, while the cut-off value for I-FABP was 12.10 pg/ml, with an AUC of 0.870, a sensitivity of 92.5 %, and a specificity of 72.6 %.

### HMGB1 is a risk factor for mortality in neonatal NEC patients

Finally, the clinical characteristics between survival and deceased NEC patients were compared. It was observed that the serum levels of HMGB1, I-FABP, IL-1β and IL-6 were markedly higher in deceased patients, when compared to survival patients (all, *P* < 0.05; Table 3). The logistic multivariate regression revealed that I-FABP, IL-1β and IL-6 are independent risk factors for mortality in neonatal NEC patients (Table 4).

**Table 3:** Comparisons between survival and deceased patients in NEC patients

Variables	Survival, *n* = 87	Deceased, *n* = 19	*P*-value
Age at diagnosis, weeks	45.79 ± 24.36	57.10 ± 22.25	0.066
Gender, female (%)	38 (43.68)	7 (36.84)	0.324
Gestational age, weeks	30.97 ± 4.97	31.36 ± 4.57	0.753
Birth weight, kg	2.77 ± 0.76	2.64 ± 0.73	0.497
1-min Apgar score	6.98 ± 1.38	6.89 ± 1.41	0.790
5-min Apgar score	8.11 ± 1.36	7.78 ± 1.39	0.351
Delivery mode, *n* (%)			0.177
Vaginal delivery	54 (62.06)	10 (52.63)	
Caesarean delivery	33 (37.93)	9 (47.37)	
Surgery methods, *n* (%)			0.128
Laparotomy	41 (47.13)	11 (57.89)	
Peritoneal Drainage	46 (52.87)	8 (42.11)	
HMGB1, pg/ml	133.06 ± 59.41	445.29 ± 168.37	< 0.001
I-FABP, ng/ml	17.99 ± 4.42	34.65 ± 9.06	< 0.001
CRP, pg/ml	492.02 ± 179.51	482.41 ± 164.81	0.831
IL-1β, pg/ml	70.22 ± 21.14	118.86 ± 45.22	< 0.001
IL-6, pg/ml	78.63 ± 33.00	131.21 ± 55.80	< 0.001
TNF-α, pg/ml	458.62 ± 177.42	505.81 ± 180.03	0.297

**Table 4:** Risk factors associated with mortality in NEC patients by logistic multivariate regression analysis

	Wald	Odds ratio	95 % CI	*P*-value
Increased HMGB1	2.603	0.173	1.188 (0.963 ~ 1.466)	0.106
Increased I-FABP	14.159	0.366	1.443 (1.192 ~ 1.746)	< 0.001
Increased IL-1β	16.517	0.052	1.054 (1.027 ~ 1.081)	< 0.001
Increased IL-6	15.272	0.032	1.032 (1.016 ~ 1.049)	< 0.001

## Discussion

NEC is the most serious intestinal disease in neonates. Its incidence is gradually increasing, and its mortality rate remains high. The early diagnosis of NEC is important for early treatment and better prognosis. Thus, novel diagnostic biomarkers with high sensitivity and specificity are needed. In recent years, HMGB1 has been frequently observed in inflammation response and related diseases. However, the clinical significance of HMGB1 in neonatal NEC patients remains unclear. The present study demonstrated that the serum levels of HMGB1 increases in neonatal NEC patients, and are correlated with the serum levels of I-FABP, IL-1β and IL-6, as well as the patient’s prognosis, indicating that HMGB1 might be used as a diagnostic and prognostic biomarker for NEC patients.

The present study initially presented the upregulated serum levels of HMGB1 in NEC patients. The role of HMGB1 in inflammation has been illuminated in various studies, including NEC and experimental colitis. Yu et al. reported that the suppression of HMGB1 can lead to the inhibition of experimental NEC through the inhibition of NLRP3 [14]. It was also found that melatonin can improve DSS-induced colitis through the suppression of HMGB1 in intestinal tissues. [15] In a clinical study, it was found that the fecal HMGB1 expression significantly increases in pediatric and adult patients with Crohn’s disease and ulcerative colitis [16]. Wazea et al. also reported that galantamine can inhibit the colitic effect through the regulation of NF-κB/HMGB1/RAGE signaling [17]. In the present study, it was found that HMGB1 was elevated in neonatal NEC patients, especially in patients with stage II-III NEC, and in deceased patients, when compared to suspected stage I NEC patients and healthy controls. Hence, HMGB1 can potentially be used as a new diagnostic biomarker for NEC. HMGB1 is an inflammatory factor, which may induce the release of other inflammatory factors, and may activate inflammation response, including pyroptosis. Thus, the elevated HMGB1 might imply that HMGB1-mediated inflammation signaling pathways are also activated in the NEC process. However, further studies are needed to confirm this hypothesis.

In the present study, it was found that inflammatory factors were overexpressed in NEC patients, and were correlated with HMGB1. Inflammatory factors, as well as I-FABP, have been proven to be associated with NEC and colitis development. Coufal et al. reported that NEC infants have a remarkably higher urinary expression of I-FABP, and that infants with higher I-FABP have a higher risk to develop sepsis [18]. It was also found that calprotectin and I-FABP levels are positively correlated with the NEC stage [19]. Factors CRP, IL-1β, IL-6 and TNF-α were also found to be overexpressed in NEC or experimental colitis [9, 10, 20]. In the present study, it was observed I-FABP and inflammatory factors CRP, IL-1β, IL-6 and TNF-α were all elevated in NEC patients. Furthermore, I-FABP, IL-1β and IL-6 were positively correlated with HMGB1 in NEC patients. However, deeper insights are needed to reveal the underlying molecular mechanisms between HMGB1 and these inflammatory factors.

The present study has some limitations. The study sample was small, and the relationship between HMGB1 and I-FABP, as well as HMGB1 and CRP, IL-1β, IL-6 and TNF-α, was not investigated. Furthermore, the gestational age was older in healthy controls, which was partly because NEC often appears in premature infants, especially in neonates with low body weight. Thus, the relationship between the levels of inflammatory factors and HMGB1, and gestational age might have influenced the results. More clinical and *in vitro* studies are needed to clarify these findings.

## Conclusions

In summary, this observational study revealed that serum HMGB1 levels are upregulated in neonatal NEC patients, and are correlated with the patient’s prognosis. Hence, the serum HMGB1 level can be potentially used as a diagnostic biomarker for NEC. The present study provides clinical evidence and basis for the application of HMGB1 as a diagnostic and prognostic biomarker for NEC patients.

## Data Availability

The data and materials are available from the corresponding author upon reasonable request.

## Abbreviations

NEC: Necrotizing enterocolitis
ELISA: Enzyme-linked immunosorbent assay
CRP: C-reactive protein
I-FABP: Intestinal fatty acid binding protein
HMGB-1: High mobility group box-1
ANOVA: Analysis of variance
